# Benefits and Challenges of a Digital Exercise and Mind-Body Program During Active Cancer Treatment: Qualitative Study of Patients’ Perceptions

**DOI:** 10.2196/80075

**Published:** 2026-01-16

**Authors:** Karolina L Bryl, Sierra Silverwood, Krupali Desai, Kelsey Schobert, Xiaotong Li, Susan Chimonas, Jun J Mao, Erin F Gillespie

**Affiliations:** 1 Integrative Medicine & Wellness Service, Department of Medicine Memorial Sloan Kettering Cancer Center New York, NY United States; 2 Department of Radiation Oncology University of Washington School of Medicine, Fred Hutch Cancer Center Seatttle, WA United States; 3 Department of Epidemiology and Biostatistics Memorial Sloan Kettering Cancer Center New York, NY United States; 4 Department of Radiation Oncology Memorial Sloan Kettering Cancer Center New York, NY United States

**Keywords:** integrative oncology, integrative medicine, digital health, supportive cancer care, mind-body therapies, exercise, qualitative research, symptom management, telehealth

## Abstract

**Background:**

Individuals undergoing cancer treatment often face a high symptom burden that impairs quality of life. Exercise and mind-body therapies have been shown to reduce symptoms but are underused. We developed a digital exercise and mind-body therapy program that effectively reduces symptoms while overcoming in-person delivery barriers. Understanding patient experiences can inform treatment mechanisms and guide digital health interventions in cancer care.

**Objective:**

This study aimed to explore patient experiences with Integrative Medicine at Home (IM@Home), a 12-week live digital program delivering exercise and mind-body therapies tailored to the needs of individuals undergoing cancer treatment.

**Methods:**

This qualitative study was embedded in a randomized clinical basket trial (NCT05053230) evaluating the effects of IM@Home versus enhanced usual care on symptoms and acute health care utilization in adults with solid tumors undergoing active treatment and experiencing moderate or greater fatigue. Using maximum variation sampling, 20 participants were selected for semistructured interviews. Interviews explored participants’ experiences with the program, its impact on outcomes, unmet needs, and suggestions for improvement. Transcripts were analyzed using a combined inductive and deductive thematic analysis.

**Results:**

Twenty participants (mean age 63, SD 9.6 years; 18/20, 90% female) were interviewed. Five major themes emerged: (1) IM@Home alleviated symptom burden and supported symptom self-management; (2) IM@Home facilitated social support and information exchange; (3) IM@Home offered a flexible, tailored program in a group setting; (4) IM@Home facilitated accessible, cost-effective support; and (5) recommendations for program enhancement. IM@Home was perceived as an accessible, flexible, and supportive program that promoted physical and emotional well-being during treatment.

**Conclusions:**

IM@Home demonstrates a promising model for delivering integrative supportive care during cancer treatment. Findings highlight patient-valued features such as real-time guidance, tailored content, and community support. These insights can inform future implementation, integration into clinical care, and efforts to enhance digital mind-body interventions in oncology.

**Trial Registration:**

ClinicalTrials.gov NCT05053230; https://www.clinicaltrials.gov/study/NCT05053230

**International Registered Report Identifier (IRRID):**

RR2-10.1038/s41746-024-01387-z

## Introduction

In 2024, an estimated 2,001,140 new cancer cases were expected in the United States [1], with most individuals undergoing systemic therapy or radiation [2]. While these treatments have significantly improved survival rates, people with cancer often experience a high symptom burden, including fatigue, pain, and insomnia, which can lead to reduced quality of life and, in some cases, treatment discontinuation [3,4]. A recent systematic review found that up to 90% of patients undergoing active cancer treatment reported unmet needs, with psychological (eg, anxiety and fear) and physical (eg, tiredness and lack of energy) challenges being most prevalent [5]. Exercise and mind-body therapies (eg, meditation, yoga) are endorsed by leading oncology organizations, including the American Society for Clinical Oncology, the Society for Integrative Oncology, and the National Comprehensive Cancer Network, as effective strategies for managing cancer treatment-related symptoms [6-10], therefore, improving treatment adherence and survival rates [11-13]. However, clinical implementation remains limited due to barriers, such as limited access, restricted modality options, and logistical challenges (eg, travel) [14-19]. Innovative delivery methods are urgently needed to address these barriers and integrate these therapies into oncology care.

Digital health technologies, including remote delivery of clinical or supportive care via telecommunications (telehealth), health services delivered through mobile health devices, and internet-based health services (eHealth), show promise for improving access [19-22] and are widely used in cancer care for symptom management [23-27], treatment supervision [28,29], and clinical trials [30-33]. Examples relevant to cancer care include mobile apps for symptom tracking and medication [34,35] and wearable activity monitors to promote physical activity and exercise [36,37]. However, their adoption for delivering exercise and mind-body therapies during cancer treatment remains limited, with concerns including ensuring safety when patients engage remotely, tailored patient-centered program design for online group formats, and addressing technical barriers [19]. To address these gaps, at Memorial Sloan Kettering Cancer Center (MSK), we developed a novel Integrative Medicine at Home (IM@Home), a 12-week live, digital telehealth program delivering group-based exercise and mind-body therapies [38,39], designed specifically for individuals with cancer. IM@Home combines multiple evidence-based modalities—including yoga, meditation, and fitness—with intensity and activities tailored to participants’ needs and treatment-related limitations. Patients have unlimited access to classes, allowing them to choose sessions based on their preferences, providing flexible and ongoing support. A randomized clinical trial (NCT05053230) of 200 patients in active treatment demonstrated IM@Home is safe and effective in reducing fatigue, anxiety, and depression, while lowering acute health care utilization [40].

While quantitative data support IM@Home’s clinical benefits, understanding the end user experience is essential to inform broader implementation and patient-centered refinement, particularly in oncology, where patient safety and care needs are heightened [41-43]. To fully understand how complex interventions such as IM@Home lead to change in outcomes, it is important to examine the broader context in which these interventions operate. This includes moving beyond standardized outcome assessments to actively include the voices of individuals directly targeted by the intervention, whose experiences and perspectives are crucial to refining the delivery and ensuring real-world relevance [44]. Yet, few studies have examined patient perspectives on synchronous digital mind-body interventions during active treatment, a period marked by significant physical, psychological, and logistical challenges [41,43]. This study addresses that gap by exploring participants’ views on the program’s digital delivery, usability, and impact on symptom relief and quality of life. The findings offer insights to support the safe, effective, and accessible integration of digital integrative oncology interventions into routine cancer care.

## Methods

### Study Design

This qualitative study was conducted as part of the Integrative Medicine for Patient-reported Outcomes, Values, and Experience (IMPROVE; NCT05053230) trial, a randomized clinical basket trial assessing the effects of the IM@Home program on symptoms and acute health care utilization in patients with moderate or greater fatigue undergoing active treatment for melanoma, head and neck, lung, gynecological, or breast cancer. From October 2021 to March 2023, a total of 200 participants were randomized to 12 weeks of either IM@Home intervention (live exercise and mind-body classes via Zoom [Zoom Video Communications]) or enhanced usual care (standard of care plus on-demand meditation resources). Study design, primary results, and detailed program description have been published elsewhere [38,40].

The IM@Home program offers 23 live classes per week, including movement-focused classes (eg, fitness and dance), mind-body classes (eg, yoga and tai chi), and mind-focused classes (eg, meditation and music therapy). Classes are delivered synchronously (in real time) via Zoom, each lasting 30-60 minutes, and led by licensed MSK Integrative Medicine Service clinicians—including certified fitness trainers, yoga and mindfulness instructors, and licensed music therapists with experience in oncology—who guided participants, offered modifications, and provided verbal cues and encouragement to support engagement and safety. Upon randomization, a member of the research team contacted participants by phone to introduce the program, provide an overview of class types, and assist with registration. Participants could attend as many classes as desired each week, select any combination of classes, and modify their selections as needed.

### Participants

We used maximum variation sampling, targeting approximately 20% of IM@Home participants to capture diverse experiences across cancer types and different stages of program participation (study time points: Weeks 4-7, Weeks 8-12, or after study completion). The sampling framework, including the number of participants selected from each cancer type and time point, can be found in Multimedia Appendix 1. Selected participants were contacted by a member of the research team via phone to invite them to participate in the qualitative interviews. All participants approached for interviews agreed to participate.

### Data Collection

We conducted semistructured interviews with 20 IM@Home participants, led by 2 trained qualitative researchers with backgrounds in integrative therapies and psychology (KS and KB). Interviewers had previously briefly interacted with some participants during their participation in the IM@Home program, either during consent or later while providing assistance with class registration. At the start of each interview, participants were informed of the interviewers’ professional roles. The research team developed and used an interview guide to explore key aspects of participants’ experiences while allowing flexibility in responses (Multimedia Appendix 2). To ensure participant-driven insights, interviewers used open-ended probing questions and adapted them in real time to explore emerging topics.

Interviews were scheduled according to participant preference and availability, conducted via HIPAA (Health Insurance Portability and Accountability Act)-compliant Zoom, lasted up to 20 minutes, and were audio-recorded with the participant’s consent using the Zoom recording function. Audio files were securely stored on an encrypted, password-protected institutional server accessible only to the study team. The audio recordings were transcribed verbatim (KS), reviewed for accuracy (KB), and de-identified by both coders (KS, KB) prior to analysis. Reflexive journaling and team debriefings were used to minimize interviewer bias.

### Data Analysis

We analyzed all interview transcripts using a 2-phase approach that combined inductive and deductive thematic content analysis [45,46]. Our analytic process followed the 6-phase reflexive thematic analysis framework described by Braun and Clarke [46] familiarization with the data; generating initial codes; searching for themes; reviewing themes; defining and naming themes; and producing the final report.

In Phase 1, two researchers (KS and KB) independently read 5 transcripts to identify key narrative content and determine the unit of analysis (eg, sentences or full passages) (Phase 1: familiarization). Through an inductive, open coding process, they developed descriptive and interpretive codes (Phase 2: generating initial codes), allowing themes to emerge from the data. The researchers then met to discuss coding and emerging themes and iteratively refined and grouped codes into higher-order categories, forming thematic units (Phase 3: searching for themes; Phase 4: reviewing themes). Analytic memo writing supported the identification of emerging patterns and conceptual relationships. This iterative process ensured that findings were grounded in participant experiences rather than predetermined categories.

In Phase 2, using a deductive approach, the finalized coding scheme was applied across all transcripts by 2 researchers (KS and KB) to assess coding consistency and intercoder agreement. A third researcher (KD) reviewed coded transcripts to enhance credibility, with any discrepancies resolved through discussion. Once coding was completed, the final themes, categories, and representative quotes were reviewed and discussed with the broader research team (Phase 4: reviewing themes**;** Phase 5: defining and naming themes; and Phase 6: producing the report). To further enhance confirmability, an independent reviewer (SC) examined the coding and interpretation for potential bias or inconsistency. NVivo software (version 14; QSR International) was used to support data management and analysis. Thematic saturation was evaluated continuously throughout the analysis.

### Strategies to Ensure Trustworthiness

Credibility was supported through researcher triangulation (3 coders participated in data coding, analysis, and interpretation), peer debriefing with the broader research team, and reflexive memo writing. Transferability was addressed through purposeful sampling and thick description of the context, participants, and findings. Dependability was ensured via a detailed audit trail documenting all stages of data collection, coding, and analysis, along with intercoder agreement among coders.

Confirmability was enhanced by maintaining an audit trail, practicing reflexivity, and conducting an external audit by an independent reviewer (SC) to assess for bias or inconsistency.

### Ethical Considerations

The study was approved by the Institutional Review Board (IRB) at MSK (IRB# 21-369), registered at ClinicalTrials.gov (NCT05053230). The ethics approval for the qualitative interviews was part of the original IRB approval. Participant confidentiality was maintained; data were collected and stored in secure, password-protected systems and analyzed in deidentified form. Participants did not receive compensation for study participation. All participants provided written informed consent. Findings are reported per the Standards for Reporting Qualitative Research guidelines [47] and COREQ (Consolidated Criteria for Reporting Qualitative Research) extension [48]. The COREQ checklist is provided as a Multimedia Appendix 3.

## Results

### Participant Characteristics

All participants approached for interviews agreed to participate. Of the 20 participants interviewed (mean age 63, SD 9.6 years), a majority were female (18/20, 90%), White (17/20, 85%), and had breast cancer (9/20, 45%). Table 1 summarizes the demographic and clinical characteristics of the 20 participants included in the qualitative interviews. This qualitative study sample was broadly reflective of the overall IMPROVE trial population (N=200) and the IM@Home arm (n=99) with respect to gender, age, and cancer type, although there was a slightly higher proportion of White and Asian participants and a slightly lower proportion of Black participants compared with the IM@Home arm.

**Table 1:** Participant characteristics.

Participant characteristic		Values
Age (years), mean (SD)		63 (9.6)
**Gender, n (%)**		
	Female	18 (90)
	Male	2 (10)
**Race, n (%)**		
	Asian	2 (10)
	Black	1 (5)
	White	17 (85)
**Ethnicity, n (%)**		
	Non-Hispanic or Latino	18 (90)
	Hispanic or Latino	0 (0)
	Unknown	2 (10)
**Cancer type, n (%)**		
	Breast	9 (45)
	Gynecological	3 (15)
	Head and neck	4 (20)
	Lung	3 (15)
	Melanoma	1 (5)
**Time of the interview, n (%)**		
	After study completion	2 (10)
	Week 4-7	7 (35)
	Week 8-12	11 (55)

### Qualitative Findings

Saturation was achieved by the 17th transcript; however, all 20 interviews were coded to capture potential nuances and ensure comprehensive representation. Ultimately, 5 major thematic units were identified. These thematic findings are summarized in a narrative account below, while detailed theme descriptions, including definitions, categories, subcategories, and additional illustrative quotes, are provided in Table 2.

**Table 2:** Qualitative findings.

Themes and their definitions, and categories and subcategories			Example quotes
**Theme 1. IM@Home alleviated symptom burden and supported symptom self-management:** *IM@Home helped participants manage cancer-related symptoms by improving physical strength, reducing fatigue, alleviating stress and anxiety, and providing accessible self-coping strategies to enhance overall well-being.*			
	Reduced treatment-related symptom burden	“The thing that definitely did help was also, you know, in terms of trying to sleep the guided part of getting you to relax, because I’ve always been a bit of an insomniac, and I think this one didn’t. This whole episode of going through all this didn’t help in that one. So that part definitely helped just being able to kind of visualize every part of you, relaxing and going to sleep.” [P003] “It’s hard to explain, I know it’s good for me and I do it. My back hurts and my neck hurts, and whatever I’m doing…I don’t know if it’s the Chair yoga or one of the other things, but my back feels better. The neck, I think, got a little worse. But the back feels better, so I know, whatever I’m doing it is helpful in the long run.” [P002] “I really like it. I feel like a different person. I feel so much looser. I just feel like ‘wow’, and it’s not like you’re sweating, and it’s really tough, but you can really feel these small movements, and I just feel good. I feel so much better like it gives me quite a bounce to my day.” [P017] “I do feel that it was um I do feel like it did give me a greater range of motion, and I do feel that I slept better.” [P010]	
	Enhanced symptom self-management and sense of control	“I would describe it [IM@Home program] as a tool for coping. Because I think being able to figure out how to um move in the world emotionally and physically with the illness, or the recovery from an illness is coping and coping and adapting is critical.” [P010] “I think it’s such a stressful time in your life, because you really don’t know what to what to expect. What you’re going through and all, you don’t know how you’re physically the physically going to be reacting to anything, and it’s such a stressful time. I think if you can plug that [IM@Home] into your day in some way, even, maybe a couple of times a day to help you kind of get yourself grounded, and I think that that would be helpful. I think you just have to try and stay focused as much as you can, even though it’s quite difficult.” [P011] “I definitely feel more like myself […] where I’m not so consumed with cancer and treatments, like I almost feel like I can. I feel mentally better. That’s one thing I would definitely say, I don’t feel as upset all the time. I don’t feel as down, because it was definitely getting, like, as the as the radiation went through like definitely emotional and teary and sort of feeling sorry for myself. So I definitely feel that I do feel more positive and just mentally like a bit more clear and like a bit more positive and going forward.” [P017] “Yes, actually, psychologically better because I finished it. Finally, you got up off the couch from watching TV or something on a day when I was doing that. Not as good as I felt if I could get out shopping for three hours. That is the ultimate kick in the pants. Yeah that [IM@Home] gets me going.” [P005]	
**Theme 2. IM@Home facilitated social support and information exchange:** *the program fostered a sense of community, offering emotional support, reducing isolation, and enabling peer-to-peer learning through shared experiences and instructor guidance.*			
	Building a sense of community	“There’s also a sense of community because usually in the beginning, at the end there’s little discussion, a talk, and you hear people talking giving little advices, there is a little community there and I like it.” [P004]	
	Feeling seen and supported	“Beyond the exposure to something that can provide comfort and health benefits, there is a certain companion ability among people if you attend the same class. There are some of the same people in those classes, and they do talk to the instructor primarily after the class. […] And you know, in the chat function afterwards, somebody will mention [personal stories] and just a nice kind of things that people would say, or someone talks about something they’re having a hard time with, or something that went well. And there are all these other people don’t know, but who are saying, you know congratulations, or you know that’s tough, or maybe throw out a resource they’ve heard of. So it’s not a big bulletin board, it’s not a big chat, but there’s a little bit of a sense of community I think, is nice.” [P012]	
	Knowledge and resource sharing	“I think it’s very good for that [sharing personal experiences] it seemed like a lot of the conversations and the chats and the things that they [patients] were talking to each other. You know this is the best speaker [platform] to use. You know, I think, all that neighborliness is very useful too. I think a sense of community is always good.” [P010] “I don’t have my camera on but some people do and it builds a camaraderie, which some people might need. Thank God, I have a lot of support from friends, from family members, you know, but some people might not have that. And to get a support from the group of people, I think that that would be very beneficial.” [P002]	
**Theme 3. IM@Home offered a flexible, tailored program in a group setting:** *participants valued the program’s safe, flexible, and personalized approach within a group format, allowing them to engage in oncology-tailored exercises suited to their needs, schedules, and symptom burdens.*			
	Ensuring safety and personalization	I think the most helpful was just the fact that the instructors were clear […] they made it very clear like ‘Well, here’s our warm-up,’ and they really did make sure, like I’ve been to some fitness classes where there’s really no emphasis on the warm-up or on the stretching afterwards. And that’s such an important part of the program. So I thought that that was probably the most beneficial. And, in my opinion, is that they really did look at this holistically and say, like, here are people who are recovering and or bring in something so let’s make sure that they stretch appropriately before we dive into the program, and then let’s make sure at the end.” [P020] “Well, I’ve been sick, for almost two years, I’m getting better now, and it makes me feel good. I mean after yoga, gentle yoga it’s very gentle but it’s just my speed, my level and I can’t believe how good it is for me and then like its wow that feels so easy, then I feel the stretches, I feel the muscles that I haven’t used for a while. So [the classes] make me feel good.” [P004] “There was a large group of people of varying levels. And so instructors wanted to make sure that everybody was able to participate. I like the emphasis on breathing. I like the emphasis on just moving, being able to do what you know. Just move. Just do whatever you can to move.” [P020] “It’s something that’s very good for you, and you don’t need like a lot of equipment, either, because that’s the other thing that people find discouraging is oh, like I don’t have weights. Well, you could use water bottles, or you could use towels, or you could use. I think what she said was, you could use your thumb and your index finger, like those types of modifications, were very helpful. So I think that’s how I would sell it to her judgment-free, all activity levels the privacy of your own home with instructors that give you different options for your fitness, level.” [P010]	
	Offering variety and flexibility	“There are abundant resources through the program for it [symptoms], access to, particularly to mind, body experiences that are interesting to explore if you’ve never done them before, and if you've done them before they’re a way to maintain a practice […] It’s good for you. It feels good to do it, and it can help you step outside yourself a little bit.” [P012] “You can choose which ones you want, and there’s a variety and different levels.” [P015] “I think that’s what I liked best about this program that it was just at a level and at a pace where you can kind of ease into it and then modify as you got stronger.” [P020] “I didn’t find it difficult or challenging, because I think people provided enough guidance to say, ‘Hey, here, if you are a beginner, here’s where you could do it.’ This is, for example […] push-ups right, you could use a wall or a chair or the floor or do a full-on push-up. So, there’s different types of push-ups you can do depending on your level. I didn’t really think it was all that challenging. And then there was always the caveat that if you weren’t comfortable doing that, then, you know, and just keep moving or do something else.” [P020] “It was a good variety. It gave you just different the different chances to do the different types of classes. Also, for the length of time that I had on my schedule so that I was able to do it. I thought the variety was good. Having them [classes] at the different time sequences, also the different types they had, where they kind of had you going you know mentally and all that. That was also very good, because it gave some variety.” [P011]	
	Role of instructors	“And the most helpful is because the instructors also say you can do this, keep going and they motivate you to go, and that helps me. It’s just a way that they, I can’t explain it, it’s just the way that they do the classes that I enjoy and it’s like, you know, like when I get tired, I just keep going.” [P018] “I think the other positive was that there was pretty good instruction, like sometimes when you do online classes. and you know, and the cameras aren’t turned on for most of the people like you don’t know if they’re going to do something that might injure themselves, but they manager themselves. But I feel like people were, you know, in a pretty good... they gave pretty good instruction. But I know that’s always a challenge with online classes, especially when you’re when you have a group of people of varying levels. Making sure that no one gets hurt.” [P020]	
**Theme 4. IM@Home facilitated accessible, affordable, and ongoing support:** *the digital format removed barriers such as cost, travel, and infection risk, making exercise and mind-body therapies accessible to diverse patients while supporting long-term wellness beyond active treatment.*			
	Improving accessibility and convenience	“I would be concerned about going to the gym and even though that don’t have to have a mask for indoors now I’ll continue wearing a mask because I don’t need to catch anything else, and a lot of people that are basically homebound at this point either because of the situation, because of COVID, or because of the way they feel. It’s good to have something that they could do at home due to their fitness level. That’s not difficult and is enjoyable. I would recommend it.” [P002] “Especially after surgery and radiation, when you’re tired and you want to regain your energy, it’s most people aren’t self-motivated to join a gym or do anything like that, but just the fact that it’s in privacy of your home that there are people that are there that are just like you. And that it’s geared and tailored for patients.” [P020] “I was a very regular gym goer and I always would go in, lift weights and do some cardio, and would always take a yoga class. I probably did like 4 or 5 a week […] since the pandemic, I’d stopped going to the gym, for fear of infection. And because of the pandemic I’m still not there, I’m not going to a gym. I’m just not going to be in a big building with people breathing hard and no masks. So that that’s the biggest benefit that it’s just the ability to get back into some sort of fitness routine.” [P015] “It was simple. I mean I just chose a class clicked on it got a registration form put in my name, address phone number whatever and then the next day or I got a link to the class the simple.” [P002]	
	Providing structure and motivation	“Sometimes the treatment can have a very demotivating effect, and having something to look forward to, I feel helped so much, mentally.” [P016] “Even though I get tired I can keep going better than before I was doing the classes, so I really think that they’re starting to help build me up again, […] which is a good thing, because I was almost to the point where I was just sitting in my chair and said, ‘Forget this, I’m tired’ So now I’m actually looking forward to doing the classes.” [P018] “And the most helpful is because the instructors also say you can do this, keep going and they motivate you to go, and that helps me. It’s just a way that they, I can’t explain it, it’s just the way that they do the classes that I enjoy and it’s like, you know, like when I get tired, I just keep going.” [P018]	
**Theme 5. Recommendations for program enhancement:** *participants provided several suggestions for optimizing the program’s implementation and maximizing its benefits.*			
	Need for additional guidance	“I think the one thing that can be a little difficult, which maybe in-person would have helped, is the Tai chi. I tried doing it, and it’s so hard to, because you have like a mirror image, and the instructor is telling you to twist your body a certain way. It’s hard to figure out exactly how to do that. So I think that was the only class that I was very confusing in terms of doing it online.” [P016] “One thing I will say about the course description, that I read before I went to each class, was I didn’t feel that they were very clear on the type of material you should bring to the class […] I don’t recall now what they said but it’s like some classes, we use stretchy bands and I know [instructor], would talk a lot about what you could use to substitute and maybe you don’t want to discourage people from coming you know joining thinking that they don’t have all the materials that are maybe used in the class.” [P006]	
	Expanding delivery methods	“Let me just say this, if you could record the classes and rerun them in the evening, right the same class. So, for example, you know [instructor’s name] does a tai chi class in the morning, and then you ran a recording of it at night where people couldn’t attend in the morning. I mean it’s I know it’s a whole technological issue to get that set up and to do it, but you wouldn’t have the instructor having to instruct again in the evening, you could replay a class.” [P006]	
	Timing considerations for the program introduction	“Having the program being offered to patients in conjunction with we start treatment and have someone help us come up with a training and exercises plan from the beginning would help to be active.” [P008] “Why can’t the doctors mention it to the patients when they do like the five week follow up from their surgery and say, ‘Oh, we have an Integrative program. I think it would be good for you to have exercise in your healing process to recover better.’ And maybe you have to like kind of coordinate it with the doctors.” [P009]	

#### Theme 1. IM@Home Alleviated Symptom Burden and Supported Symptom Self-Management

Participants reported that the IM@Home program was valuable for managing cancer-related symptoms during treatment, offering physical, mental, and social benefits.

#### Reduced Treatment-Related Symptom Burden

Participants highlighted significant improvements in physical and mental health, including reductions in cancer treatment-related symptoms, particularly radiation-related fatigue, overall stress, and anxiety. Fatigue reduction led to increased muscle strength, flexibility, and mobility, while decreased stress and anxiety enhanced their motivation to stay active.

It really helped. I feel like my muscles are stronger, my walking is a lot easier, and I’m not as tired as I used to be.

P006

What I noticed was with the radiation fatigue, I think about week three, I was like, ‘Wow, I’m good.’ I've got my normal energy back, like there’s no more fatigue. That was probably the biggest benefit, just like flipping a switch, ‘I’m good!’

P015

These improvements collectively increased their ability and willingness to continue classes and carry out daily activities and further contributed to the reduction of comorbid symptoms such as insomnia. Yoga and fitness classes were frequently mentioned for fatigue management and meditation classes for reducing stress.

#### Enhanced Symptom Self-Management and Sense of Control

Many participants described the challenges they faced during treatment, particularly after leaving the hospital. This was when treatment-related symptoms emerged, requiring them to manage these difficulties on their own, often leading to a sense of lost control. In this context, participants noted that IM@Home provided valuable, accessible, and easy coping skills, such as breathing techniques, to help them navigate symptoms or manage difficult moments at home. Additionally, completing classes provided participants with a sense of control, giving them the courage and confidence to continue their ongoing treatments.

With all the stress of the diagnosis and treatment that’s one [breathing and relaxation] that I realized is a good thing for the stress release […] there were days when things were happening to me, and my ability to just lay there and count my breaths or breathe through things was really a great thing.

P015

The anticipation of classes also provided mental relief, with one participant noting, “Having something to look forward to helped so much mentally” (P016). Another participant highlighted the program’s impact on self-commitment and achievement.

At the conclusion of each class, there is a feeling of accomplishment. I did something for me, I kept a commitment to myself. I showed up and got through the whole thing. I feel good about doing it.

P012

#### Theme 2. IM@Home Facilitated Social Support and Information Exchange

The program fostered a sense of community, helping participants feel less isolated. As one participant noted, “There is a feeling that you’re doing this together” (P012). Another described class interactions as feeling like “a friend came into their home” (P005).

Participants valued the emotional support of being seen and not feeling alone.

You see the other people that are in the class with you, that’s comforting not to be alone, because a lot of us are alone.

P013

Receiving support and encouragement from peers and instructors contributed to a more positive experience during the treatment process.

It’s comforting seeing the instructor and hearing them acknowledge you makes a difference.

P013

Participants described how postclass discussions provided opportunities to exchange advice and learn from others’ experiences, further enriching the program’s social aspect.

I learned a lot from the instructor’s comments about the effects of radiation on muscles and fascia, which came from an exchange with another class participant […] It was enlightening for me, and it was what I was experiencing.

P012

#### Theme 3. IM@Home Offered a Flexible, Tailored Program in a Group Setting

Participants described their experience with IM@Home as safe, patient-centered, and flexible. They noted differences compared to other general fitness classes they had attended, expressing that IM@Home felt safe and comfortable. The activities were tailored to fitness levels and the unique physical and mental needs of cancer patients, including those with metastatic disease and a high symptom burden.

With good tolerance and noticeable benefits, they continued engaging with IM@Home and used it as a valuable tool for maintaining their quality of life. As one participant explained,

Exercise and [being active] can keep things from reoccurring, maximize your lifestyle and functionality, and help you regain skills you’ve lost. When you’re living with an illness, dealing with something chronically, you need as many tools as you can to alleviate pain and symptoms and to continue to live as fully as you can, even though you’re still sick.

P010

Participants also noted the key contributions of instructors specialized in oncology care.

The instructors were really top-notch, and they were specifically tailoring the exercises and the activity levels.

P010

Additionally, even in a group setting, patients felt that the program was individualized to their preferences, schedules, and daily routines. IM@Home offered a variety of classes, including yoga, meditation, and fitness, empowering participants to choose activities aligned with their preferences and physical needs. Its flexibility allowed them to continue participating in the face of such challenges as scheduling conflicts with treatment appointments, changes in symptoms or needs, or concerns about monotony.

I thought it was great that it would be in my own home, and with little to no equipment. All those things they thought were fantastic because when you have ongoing illness you there are things like side effects, where you might have to run to a bathroom and to physically be in a class it would make all those things difficult, whereas here you had some latitude as to when you could attend or leave the class or come back to the class, and so I thought I was primed for success at it.

P010

#### Theme 4. IM@Home Facilitated Accessible, Affordable, and Ongoing Support

Participants appreciated the program’s digital format for increasing accessibility in several ways: (1) compared to in-person classes, the digital format was more cost-effective, which was important given the financial burden of cancer treatments; (2) the digital format eliminated the need for travel, reducing barriers for those with mobility limitations; and (3) it minimized infection risks for patients who could not safely attend in-person sessions, particularly those who were immunocompromised. They emphasized that the digital format allowed not only the general cancer population but also those facing significant barriers to in-person attendance to receive support and stay active.

I don’t have to go anywhere, I don’t have to travel, I turn on my computer.

P003

While you’re going through this process and can’t get out and do some of the things you used to, or that you thought about doing […] This is a productive and psychologically healthy way to spend some of that extra time. It feels good to do it.

P011

Additionally, participants emphasized the importance of having resources to maintain healthy behaviors despite treatment side effects and even after completing cancer treatments. They appreciated the online program provided ongoing access, allowing patients to engage whenever needed and supporting their commitment to long-term wellness.

Knowing there is an interface out there I can go to any time, if I want to do some meditation or some light exercises like chair yoga, it really helps that there is something out there I can lean on.

P016

I was not exercising at all. I was becoming very lethargic. Having this program helped me on a daily basis. I signed up, I keep signing up for more and more classes, and it keeps me starting my day with exercise and it made me feel better.

P003

#### Theme 5. Recommendations for Program Enhancement

Participants provided several suggestions for optimizing the program. Some suggested offering more detailed guidance alongside the classes to help them understand complex movements or better prepare for class participation.

It’s so hard to figure out exactly how to do [movement]. It was very confusing in terms of doing it online.

P016

They also recommended incorporating additional delivery methods, besides live online classes, to benefit patients without reliable internet access or devices. Furthermore, participants proposed introducing the program earlier in the cancer care journey, such as during consultation, to maximize its benefits.

Could there be a consultation where, after meeting your surgeon, you also meet your oncologist and learn about programs like IM@Home to help you along the road?

P017

## Discussion

### Overview

This qualitative study explored the experiences of patients undergoing active cancer treatment who participated in a 12-week cancer-specific digital exercise and mind-body program, IM@Home. Participants described a broad range of benefits, including improved symptom management and enhanced social support, and reflected on the program’s novel, patient-centered, group-based online delivery. They also identified opportunities for improvement, such as adding real-time feedback, peer mentorship, and recorded sessions to complement live classes. Taken together, the findings suggest a preliminary program model in which digitally delivered, flexible integrative medicine programming promotes symptom relief and peer connection, mechanisms that supported patients during treatment. These mechanisms appear shaped by contextual factors (eg, treatment-related fatigue, mobility limitations, and scheduling barriers) and can inform refinements to optimize delivery. Overall, these findings provide timely insights into how live, structured, digitally delivered integrative care can address supportive care needs during active treatment and reinforce prior evidence on the value of synchronous, patient-centered digital interventions in oncology.

### Principal Findings

Exercise and mind-body therapies are well-documented for managing cancer-related symptoms [7,40,49-53] and for improving quality of life during treatment [40,54-60]. Our interviews’ findings on symptom management (ie, reduced fatigue and mental stress) align with previous trials and are consistent with results from our randomized clinical trial [40]. This suggests these symptoms may be primary targets for online exercise and mind-body therapies in cancer care. Additionally, our findings indicate that symptom relief came not only from the interventions but also from patients’ own continued efforts, as they learned self-coping strategies and received encouragement and support from other participants. Existing literature underscores the importance of self-coping in symptom management and healing [61-63], highlighting its role not only in managing symptoms during treatment but also in supporting long-term survivorship.

Our study provides valuable insights into the design and delivery of digital interventions. The increasing popularity of digital health reflects its ability to overcome traditional barriers to care such as accessibility and scheduling [64]. Remote delivery of clinical or supportive care via telecommunications (ie, telehealth) is particularly beneficial for immunocompromised patients, offering privacy and reduced infection risks [65]. However, many digital programs face implementation challenges in cancer care, such as balancing intervention effects with patients’ tolerance, delivering the program in a group setting while maintaining patient-centered care, and engaging patients in routine participation without adding to their burden [19,50,66,67]. Participants indicated that IM@Home successfully addressed these challenges by integrating multiple evidence-based therapies tailored to patients with cancer, accommodating different exertion levels to ensure safety and align with patient tolerance, and allowing flexible access to various treatment modalities. This flexibility enabled participants to select activities based on preferences, symptoms, and schedules, while maintaining regular engagement. These features enhanced safety, improved adherence, and expanded access, ultimately increasing the effectiveness of the interventions.

A distinctive feature of IM@Home was its live, group-based delivery format, which fostered community, reduced feelings of isolation, and created a safe, supportive environment, crucial for maintaining quality of life and coping during cancer treatment [68,69]. Participants emphasized the unique value of real-time interaction with instructors and peers, describing the experience as supportive and empowering. This structure stands in contrast to many digital interventions that rely on asynchronous content and may lack opportunities for interpersonal connection or individualized guidance. Social support was repeatedly cited as a motivating factor, as participants found validation in shared experiences and encouragement from others. These relational aspects, often overlooked in digital health design, are critical for sustained engagement and psychological support during cancer care [70]. This finding aligns with broader calls for a “patient revolution” in the health care system, emphasizing the need to empower patients to take a more active role in their care [71]. A key component of this shift is enabling patients to seek advice and support from others who have faced similar challenges. Based on patient feedback, they received empathy, insight, and practical strategies from both peers and instructors, fostering a greater sense of control and satisfaction with care and alleviating pressure on the health care system.

Our findings underscore the need to integrate digital exercise and mind-body programs like IM@Home into standard oncology care. Early incorporation into treatment planning, perhaps during initial consultations, could maximize benefits by establishing wellness routines before treatment-related symptoms worsen. Participants also highlighted the need for additional guidance or on-demand resources to complement online classes. Potential solutions, such as enhancing real-time feedback, incorporating peer mentorship, and providing recorded sessions, should be considered in future program development. Additionally, multicenter trials are needed to assess program effectiveness across diverse populations, alongside cost-effectiveness and scalability. Furthermore, improving patient education and clinician-patient communication about the benefits of digital interventions could promote adoption and adherence. By addressing current gaps, digital exercise and mind-body programs like IM@Home can be optimized to meet the complex needs of patients with cancer and expand access to effective, comprehensive care. These insights offer actionable strategies for future program refinement and broader clinical integration.

From an implementation perspective, IM@Home demonstrates a scalable model for integrating digital exercise and mind-body therapies into routine oncology care. By reducing barriers related to cost, travel, and immunocompromise, the program has the potential to increase access and address equity gaps in supportive care. Embedding such interventions early in the cancer care trajectory—and ensuring they are adaptable to individual needs—may help improve adherence and patient-centered outcomes and promote more equitable access to evidence-based supportive care. Future adaptations might include multilingual content, device-lending strategies, or hybrid models to serve patients with limited digital access.

### Limitations and Future Directions

This study has several limitations. First, the small, predominantly female and White sample limits the transferability of findings. While we used maximum variation sampling to capture a range of experiences across cancer types and study time points, the demographic homogeneity may have influenced the range of perspectives reported. Future research should aim to recruit more diverse participants to enhance the transferability of findings. Second, we did not include participants who dropped out or conduct interviews at earlier time points. Including these participants could have provided additional insights into barriers and facilitators of engagement. Third, although formal member checking—returning preliminary themes to participants to assess whether the interpretations resonate with their experiences—was not conducted, casual interactions with participants supported the credibility of the findings. Finally, future studies should also explore long-term outcomes and implementation strategies (eg, integration into routine clinical workflows and program scalability) to advance equitable integration of digital integrative care into cancer treatment pathways.

### Conclusions

IM@Home represents a novel, patient-centered, and scalable model for digital integrative care. Participants viewed the program as supportive, empowering, and effective in managing treatment-related symptoms. These findings lay the foundation for refining and scaling IM@Home to benefit diverse cancer populations and inform the development of future digital health interventions.

## Data Availability

The datasets generated or analyzed during this study are available from the corresponding author on reasonable request.

## Appendix

Multimedia Appendix 1 Maximum variation sampling framework for qualitative interviews.

Multimedia Appendix 2 Interview guide.

Multimedia Appendix 3 COREQ (Consolidated criteria for Reporting Qualitative research) checklist.

## Abbreviations

COREQ: Consolidated Criteria for Reporting Qualitative Research
HIPAA: Health Insurance Portability and Accountability Act
IM@Home: Integrative Medicine at Home
IMPROVE: Integrative Medicine for Patient-reported Outcomes, Values, and Experience
IRB: Institutional Review Board
MSK: Memorial Sloan Kettering Cancer Center
