# Supplementary material for: Benefits and Challenges of a Digital Exercise and Mind-Body Program During Active Cancer Treatment: Qualitative Study of Patients’ Perceptions
Source: JMIR Cancer. 2026 Jan 16;12:e80075. doi: 10.2196/80075 (PMC12859541; doi:10.2196/80075)
Supplement: Multimedia Appendix 1 [file cancer_v12i1e80075_app1.docx]

**Supplementary Material 1:** Maximum Variation Sampling Framework for Qualitative Interviews

| **Cancer Type** | **Study Time Point: Weeks 4–7** | **Study Time Point: Weeks 8–12** | **After Study Completion** | **Total per Cancer Type** |
| --- | --- | --- | --- | --- |
| Breast | 5 | 4 | 0 | 9 |
| Lung | 2 | 1 | 0 | 3 |
| Gynecologic | 0 | 3 | 0 | 3 |
| Head & Neck | 0 | 3 | 1 | 4 |
| Melanoma | 0 | 0 | 1 | 1 |
| **Total Participants for Qualitative Interviews per Time Point** | **7** | 11 | **2** | **20** |

**Notes:**

- Target = ~20% of IM@Home participants across cancer types and program time points.
- Sampling was guided by the distribution of cancer types in the IM@Home intervention arm (e.g., breast cancer was the most common diagnosis and melanoma the least), while ensuring representation across all types to capture a range of experiences.
- Sampling intentionally captured variation across diagnosis and timing of the IM@Home experience to characterize diverse perspectives.
