# Supplementary material for: Benefits and Challenges of a Digital Exercise and Mind-Body Program During Active Cancer Treatment: Qualitative Study of Patients’ Perceptions
Source: JMIR Cancer. 2026 Jan 16;12:e80075. doi: 10.2196/80075 (PMC12859541; doi:10.2196/80075)
Supplement: Multimedia Appendix 2 [file cancer_v12i1e80075_app2.pdf]

Supplement

Benefits and challenges of a digital exercise and mind-body program during active cancer treatment: A qualitative study of patients' perceptions

Contents

IM@Home Participants Interview Guide………………………………………………………... 2

Enhanced Usual Care Interview Guide………………………………………………………... 5

IM@Home Participants Interview Guide

My name is ______. I am [state your role on the study] from the Integrative Medicine Service at MSK. We are interested in learning your thoughts and experiences participating in the IM@Home program and the classes you have attended. Your feedback today will be used to help us improve the program and address future participants' needs. The interview should take about 15-20 minutes. Your participation is completely voluntary. You can choose not to answer a question and you can stop the interview at any time for any reason. I would like to ask your permission to record this interview. All audio recordings will be de-identified and transcribed verbatim, and your name will not be identified in any publications. I will only use the recording to listen back to our interview and fill in any gaps in notetaking. After the recordings are transcribed, I will delete the audio file.

Is it ok if I record our conversation?

· NO- Thank the individual for their time and end the call.

· YES- Continue with the next section.

Great! This might feel a little different than a normal conversation because I will be mostly listening and taking notes. There are no right or wrong answers to the questions that I will ask. Your participation is voluntary, so you may stop the interview at any time. Before we begin, what questions do you have?

· YES QUESTIONS [answer participants questions].

· NO QUESTIONS- Thank you, then we will proceed with the interview.

I will begin the recording now.

<u>Interview questions to guide conversation with participants:</u>

1. What are your overall impressions of the IM@Home program?
2. What was your experience like accessing the class sessions or on-demand library?

   PROMPT:

   a. Did you experience any difficulties participating in the classes?
3. What has your experience been like attending the IM@Home classes?

   PROMPT:

   a. What are your thoughts about the times that classes are offered?
   b. What did you think about the length of classes?
4. What are your thoughts about the variety of classes offered?

   PROMPT:

   a. There are a few different class options. What classes do you usually attend (e.g., fitness, meditation)? What made you interested in these specific classes?
   b. Are there any other classes that you would like to see on the schedule?
5. How do you feel after attending an IM@Home classes?
6. Since you've started the program, have you noticed any changes in how you feel?
7. What are the most or the least helpful elements of the IM program for you?

   PROMPT:

   a. Were there any specific classes that you did not enjoy? Why?
8. In your opinion what types of patients would benefit from this program?

   PROMPT:

a. Would you recommend this program to other [Lung, Melanoma, etc.] patients? How would you describe the program?
b. Do you have any suggestions for us on how we can reach a wider audience? For example, to other [Lung, Melanoma, etc.] patients.

9. Do you have any suggestions on how we can improve IM@Home program?

Is there anything that I did not ask you about the program that you'd like to talk about today?

Thank you for taking the time to speak with me!

Enhanced Usual Care Interview Guide:

My name is ______. I am [state your role on the study] from the Integrative Medicine Service at MSK. We are interested in learning your thoughts about and experiences of the meditation station that you used while part of the IMPROVE Study. Your feedback today will be used to help us improve our pre-recorded materials and address future participants' needs. The interview should take about 15-20 minutes. Your participation is completely voluntary. You can choose not to answer a question and you can stop the interview at any time for any reason. I would like to ask your permission to record this interview. All audio recordings will be de-identified and transcribed verbatim, and your name will not be identified in any publications. I will only use the recording to listen back to our interview and fill in any gaps in notetaking. After the recordings are transcribed, I will delete the audio file.

Is it ok if I record our conversation?

· NO- Thank the individual for their time and end the call.

· YES- Continue with the next section.

Great! This might feel a little different than a normal conversation because I will be mostly listening and taking notes. There are no right or wrong answers to the questions that I will ask. Your participation is voluntary, so you may stop the interview at any time. Before we begin, what questions do you have?

· YES QUESTIONS [answer participants questions].

· NO QUESTIONS- Thank you, then we will proceed with the interview.

I will begin the recording now.

<u>Interview questions to guide conversation with participants:</u>

1) What are your overall impressions of the meditation station?
2) What was your experience like accessing the meditation station?

   PROMPT:

   a) Did you experience any difficulties using the recordings?
3) What are your thoughts about the variety of recordings offered?

   PROMPT:

   a) There are a few different options. What recordings do you usually listen to? What made you interested in these specific recordings?
   b) Do you think that anything is missing from the recordings?
4) How do you feel after listening to a recording?

   PROMPT:

   a) Where there any recordings that you liked or did not like?
   b) How did they make you feel?
5) Since you've started the study, have you noticed any changes in how you feel?
6) What recordings are the most/least helpful?
7) What types of patients would benefit from the meditation station?

   PROMPT:

   a) Would you recommend it to other [Lung, Melanoma, etc.] patients? How would you describe the meditation station?

b) Do you have any suggestions for us on how we can reach a wider audience? For example, to other [Lung, Melanoma, etc.] patients.

8) Do you have any suggestions on how we can improve the meditation station?

Is there anything that I did not ask you about the meditation station that you'd like to talk about today?

Thank you for taking the time to speak with me!
